# Bifid Shape Is Intrinsic to *Bifidobacterium adolescentis*

**DOI:** 10.3389/fmicb.2017.00478

**Published:** 2017-03-21

**Authors:** Dhanashree, Sharika Rajashekharan, Balamurugan Krishnaswamy, Rajagopal Kammara

**Affiliations:** ^1^Department of Protein Chemistry and Technology, Council of Scientific and Industrial Research – Central Food Technological Research Institute, MysoreIndia; ^2^Department of Biotechnology, Alagappa Univeristy, KaraikudiIndia

**Keywords:** bifid, polymorphic, *Bifidobacterium*, intrinsic, aerophilic, microaerophilic

## Abstract

Although the genus *Bifidobacterium* was originally named for its bifid morphology, not all bifidobacterial species have a similar structure, and very few of them adopt a bifid shape under stress conditions. The exposure of respective bifidobacterial species to various conditions, such as different pH, temperatures, medium components, *in vivo* growth in *Caenorhabditis elegans*, and subculture, did not affect their diverse morphologies. Extensive scanning electron microscopy studies suggested that the bifid shape of *B. adolescentis* are maintained irrespective of the conditions. Hence, we conclude that the bifid morphology is intrinsic to *B. adolescentis*. Most bifidobacterial species are anaerobic and rod-shaped, whereas, after the first generation, they become microaerophilic or aerophilic. CaCl_2_ (treatment of *B. animalis*) signaling triggered a change from the rod shape to the bifid shape and vice versa in *B. adolescentis*.

## Introduction

The genus *Bifidobacterium* is so designated because in the reference species, one end was a rocket-shaped and the other, longitudinally split (Tissier, 1900). Various bifidobacterial species have been classified based on the nucleotide sequence encoding the 16S rRNA, Hsp60, and xylulose-5-phosphate (XFP; also called fructose-6-phosphate phosphoketolase/F6PPK). However, the lack of cutting-edge research into the taxonomy of the genus *Bifidobacterium* has meant that these species have been retained in *Bifidobacterium*, regardless of morphological differences. Because very few bifidobacterial strains have a bifid shape, even though some are polymorphic both rod-shaped and bifid-shaped species are retained in the single genus *Bifidobacterium*.

Investigators in the field, such as Weiss (1933), Blaurock (1937), Boventer (1938), Weiss and Rettger (1938), and Tomarelli et al. (1949a,b), have stressed the demand to develop optimal media for bifidobacterial s pecies. The reflection of variations in the bifid structures of *Bifidobacterium* and *Lactobacillus acidophilus* was confirmed by Herter (1907), Bliuhdorn (1910), Kendall and Haner (1924). Since then, some have suggested that *Bifidobacterium* and *Lactobacillus* are two different genera (Sundman et al., 1959). Exposure to stress conditions causes the latter to adopt pleomorphic forms, including the bifid form. These observations were confirmed by Rodella (1908), Cruickshank (1925), Roos (1927), and Synder et al. (1967) who reported that the alternative forms are uncommon and give an illusion of branching. Norris et al. (1950) confirmed that stress conditions induce the polymorphic forms of *Bifidobacterium*. However, the mechanisms underlying the bifid conformation are unknown, and there has been no account of this polymorphic/bifid morphology. Therefore, in this study, we investigated the bifid nature of *Bifidobacterium*. Studies of polymorphic bacterial morphologies by Moro (1905) and Tissier (1905) demonstrated that bifid-shaped organisms are present in the fecal material of breastfed infants. Populations of these organisms (bifid form) in the intestine represent the disease-free condition and are physiological significance. However, the cellular elements and physical parameters responsible for the upkeep of the bifid form *in vivo* and *in vitro* are yet to be determined.

According to available reports, there are about 63 different *Bifidobacterium* species. The nomenclature and taxonomy of these microbes are incomplete. Hence, it is critical to understand the diverse nature of *Bifidobacterium* and to determine the characteristic morphologies of all the *Bifidobacterium*. It is essential to determine whether this polymorphism is intrinsic to a particular species of *Bifidobacterium*. Weiss and Rettger (1938), reported that these bacteria lose their bifid structure and change to the rod-shaped form in the subculture. Moreover, before subculture, they are strictly anaerobic, whereas after subculture, they are aerobic. The mechanisms of all these phenomena are yet obscure. In this study, we examine the polymorphic morphologies of the *Bifidobacterium*, and conclude that the bifid morphology is an intrinsic characteristic of *B. adolescentis*, whereas (very few) other species, including *B. animalis*, display polymorphic behavior only under stress conditions. However, all the other bifidobacterial species maintain their rod shape, regardless of the culture conditions, and rarely adopt the bifid shape.

In the Homma et al. (1960) showed that univalent cations induce this unique phenomenon. Kojima et al. (1970) confirmed the role of monovalent cations, including sodium chloride, sodium sulfate, sodium nitrate, tribasic sodium phosphate, and sodium acetate, in the induction of the bifid form. Later, the roles of chlorides of Ca^++^, Ba^++^, Mg^++^, Mn^++^, and Zn^++^ were shown to play no role in this pleomorphism (Kojima et al., 1970). In the 1960s, glucose and sucrose were also proven to positively affect this polymorphism (Kojima et al., 1968).

Tissier (1899), *L. bifidus* was the only organism that was exhaustively dissected for its morphological variability. However, this work was incomplete because many bifidobacterial species are recognized today and many decades-old comparative morphological studies have not survived. In response to the original studies, the name ‘*Bifidobacterium*’ was suggested, but was considered equivocal, even then. In those days, fewer *Bifidobacterium* species were available, and *L. bifidus* may have been the only recognized bifidobacterial species. In 1950, 1968, and 1970, *L. bifidus* was the only strain in which this polymorphism was investigated. Kojima et al. (1970) demonstrated the role of CaCl_2_ in inhibiting various bacterial morphological forms. The comparative study (by Kojima et al., 1970) of cell wall composition of the bifid and bacilloid forms reported are clearly shown the restriction of amino acid phenylalanine and methionine to bifid form and the presence of high content of the glucose compared to bacilloid form. Surprisingly, larger peptidoglycans were found in the bacilliform, whereas the average-sized peptidoglycans occur in the bifid form. These observations also support the contention that ‘*Bifidobacterium*’ is a misnomer. Not all *Bifidobacterium* shows the bifid morphology because it is not an intrinsic characteristic, and may be restricted to very few members of the genus.

To examine the different morphologies of the species included in the genus *Bifidobacterium*, we selected several bifidobacterial groups constructed by Bottacini et al. (2014), each selected species corresponds to a particular group and subjected them to different stress conditions. The different Bifidobacterial groups were fed to the model system, *Caenorhabditis elegans* to assess their morphological changes during *in vivo* stress conditions and was characterized and analyzed through scanning electron microscopy (SEM). In this manuscript, we report our findings on the varied morphologies of the *Bifidobacterium*, detected with SEM and compound microscopy.

## Materials and Methods

Organisms: *Bifidobacterium* species, including *B. animalis, B. longum*, and *B. adolescentis*, etc., were obtained from the German collection of Microorganisms and Cell Cultures (Inhoffenstraße 7B, 38124 Braunschweig, Germany). For details, see **Table 1**. *C. elegans* and OP 50 *Escherichia coli* was a kind gift of Dr. Balamurugan.

**Table 1:** Strains used in this study.

Bacterial strain	Strain no.	Reference
1 *B. animalis* subspp. *lactis*	DSMZ 10140	Meile et al., 1997
2 *B. thermoacidophilum* subspp. *thermoacidophilum*	DSMZ 15837	Dong et al., 2000
3 *B. adolescentis*	DSMZ 20083	Reuter, 1963
4 *B. longum* subspp. *infantis*	DSMZ 20088	Reuter, 1963
5 *B. longum* subspp. *longum*	DSMZ 20219	Reuter, 1963
6 *B. asteroides*	DSMZ 20089	Scardovi and Trovatelli, 1969
7 *B. animalis*	DSMZ 20105	Mitsuoka, 1969
8 *B. breve*	DSMZ 20213	Reuter, 1963
9 *B. indicum*	DSMZ 20214	Scardovi and Trovatelli, 1969
10 *E. coli OP50*		A gift from Prof. Balamurugan

*B, Refers to Bifidobacterium.*

### Media and Culture Conditions

Commercially available media, such as MRS (Oxoid, Milano, Italy) and Bifidobacterial broth (Hi-Media Laboratories, A-516, Swastik Disha Business Park, via Vadhani Industrial Estate, L.B.S. Marg, Mumbai-400 086, India) and agar medium, were used to grow the different Bifidobacterial species. The first generation of the Bifidobacteria was cultured in MRS broth (and also in Bifidobacterial broth when appropriate) at 37°C (without shaking) in an anaerobic chamber for 36 h. They were sub-cultured and grown in aerobic condition in the second generation.

### Growing, Harvesting, and Growth Curve Studies

Bifidobacterial strains, *B. animalis*, *B. longum*, and *B. adolescentis*, were grown at different temperatures: room temperature, 30, 37, and 42°C. The bifidobacterial strains were streaked onto the MRS agar plate to produce single isolated colonies. The streaked plates were incubated at 37°C (for 36 h) under anaerobic conditions. A single colony was isolated, used to inoculate 10 ml of fresh MRS medium, and grown in an anaerobic growth chamber. An aliquot (1%) of each inoculum was subculture in 15 ml of fresh MRS medium to construct growth curves. The bacterial samples were collected every 6 h and the OD_600_ measured. All the experiments were performed in triplicates.

### Growth, Maturation, and Harvesting of *C. elegans*

Wild-type *C. elegans* were obtained from CGC and maintained on NGM at 20°C. The standard *E. coli* OP50 strain was used as a food source. Age synchronized young adult worms were used in all experiments. Synchronization was obtained through bleaching of gravid hermaphrodites to obtain eggs that were plated onto seeded plates. The young adult nematodes were harvested from NGM plates by using M9 buffer and used for experiments. Was grown on NGM agar plates and fed internationally accepted feed (the standard *E. coli* OP50 strain), as described previously (Ward, 1973). Standard techniques (Hope, 1999; Stiernagle, 1999) for the maintenance and growth of nematodes on NGM were used.

### Bacterial Strains and Growth Conditions for *C. elegans* Feeding

*Escherichia coli* OP50 was grown overnight at 37°C at 200 rpm in Luria-Bertani medium. The bacterial lawns used for the *C. elegans* studies were prepared by spreading 10 μl of the bacterial strain onto nematode growth media (NGM) agar (0.25% peptone) in 3.5-cm plates. The plates were incubated overnight in an incubator at 37°C. They were allowed to equilibrate to room temperature before they were seeded with *C. elegans* and incubated at 20°C throughout the experiment.

All the bifidobacterial strains cited in the **Table 1** (except *E. coli*) were grown as shown above. Bacterial lawns were made by spreading 10 μl of the bifidobacterial strains onto MRS agar in 3.5-cm plates. A similar procedure to that described above was followed, but *C. elegans* was fed different Bifidobacterial (*B. longum* and *B. adolescentis*) strains rather than *E. coli* OP50. *B. longum* and *B. adolescentis* were considered further for these studies as it is preferred by the *C. elegans*. The second generation of this bacteria is microaerophilic therefore they can grow in aerobic condition, where *C. elegans* also grows.

### Chemotaxis Assay

To determine whether the *Bifidobacterium* and their products (ocins) are non-toxic and safe for *C. elegans*, several experiments were performed (Brenner, 1974). The procedure follows as below. A Petri plate was divided into four parts, two opposite and one adjacent part labeled “Test”(A, B, and D) and the remaining as “Control” (C). All the sectors (A, B, C, and D) were equidistant from one another. Part A contained *B. animalis*, part B *B. longum*, part C the control *E. coli* OP50, and part D *B. adolescentis*. Approximately, 25 wild-type *C. elegans* that had been grown on *E. coli* OP50 were washed thoroughly and introduced into the center of the plate and the origin was marked with a circle to allow non-motile worms to be eliminated. The plates were incubated under optimal conditions, and analyzed the following day. All the bacteria were equidistant from the center where the worms started their journeys. The numbers of worms in each bacterial sector were scored after 12 and 24 h and the chemotaxis index (CI) was calculated by using the following formula.

CI=Worms on test bacterium−Worms inE.coli⁢  OP50 Total number of worms

### Phenotypic Observations of *Bifidobacterium*-Fed *C. elegans* and *C. elegans* as a Model Organism to Understand the Polymorphic Morphologies of *Bifidobacterium*

*Caenorhabditis elegans* was used as a model organism to determine the ability of the nematode to consume *Bifidobacterium* and to understand the intrinsic morphology of *B. adolescentis*. Different *Bifidobacterium* (*B. animalis*, *B. longum*, and *B. adolescentis*) were grown in the appropriate medium, as described above. They were harvested, washed, and resuspended in the appropriate medium. *C. elegans* were fed with different ratios (1:10, 1:20, and so on) of *Bifidobacterium* and left for 72–96 h. They were harvested at different intervals (after 12, 24, 32 h, and so on) without rupture, and then smashed so that the internal microbes could be observed. The worms were washed, fixed with glutaraldehyde, after a series of wash animals were crushed on the slide and analyzed with SEM. The phenotypic changes in the nematodes fed *Bifidobacterium* were observed microscopically. The cells of *B. adolescentis*-fed worms were also lysed, and the supernatants observed with SEM and the bacteria were quantified (CFU).

### Bifidobacterial (*B. longum* and *B. adolescentis*) Colonization of the *C. elegans* Digestive Tract

The method of Garsin et al. (2001), with minor modifications, was used to estimate the approximate numbers of bacterial cells in the worms. Ten worms were selected, and the surface bacteria were removed by washing the worms 4–5 times with 5 μl of M9 buffer on agar plates. Each worm was transferred to a 0.5 ml centrifuge tube containing 20 μl of M9 buffer and mechanically disrupted with a Microtube pestle (Scientific Specialties, Inc., Lodi, CA, USA). The volume was then adjusted to 100 μl with M9 buffer (serially dilute and spread) and the numbers of bacteria [colony-forming units (cfu)] were estimated by spreading the culture on MRS agar plates. The plates were incubated at the appropriate temperature until the colonies were observed. The same colonies were washed well with M9 buffer and immediately fixed and observed with SEM. Genomic DNA was isolated from some bacteria and subjected to 16S rRNA amplification and alignment.

### Understanding the Bifidobacterial Morphology through SEM

Scanning electron microscopy studies were conducted to understand the diverse morphology of *Bifidobacterium*. Various *Bifidobacterium* species were subjected to different conditions, including different media (MRS or *Bifidobacterium* broth) and different temperatures (in MRS at room temperature, 30, 37, or 42°C for SEM and Growth curve studies). They were also grown in MRS medium (for three generations also), with or without amino acids (667 mg/lt) (alanine + asparagine + glutamine + serine, or individually with alanine, asparagine, tryptophan, etc.) in the presence and absence of CaCl_2_ (Husain et al., 1972), and on MRS with and without salts (0.2 M Na_2_SO_4_, 0.2 M NaCl, or 0.2 M CH_3_COONa) (Kojima et al., 1968, the effect of salt concentration studies) and at different pH (5–7.5). The pleomorphism of Bifidobacteria was understood by growing them *in vivo* (in *C. elegans*).

### The Procedure Followed for SEM

The various bifidobacterial strains were harvested, and washed twice with phosphate buffer. The pelleted bacteria were fixed in 2% glutaraldehyde and incubated at 4°C 1 h/overnight. The cells were then harvested and washed with a 10–100% gradient of ethanol. The cells were resuspended in 50–100 μl of absolute alcohol. Approximately 2 μl of the sample was placed on a cover slip, dessicated and examined with SEM. The images were collected at 20,000 magnifications. Only appropriate and convincing images were considered in the analysis.

### Genomic DNA Isolation

The commercial GeneJET Genomic DNA Purification Kit (Fermentas, Inc., 830 Harrington Court, Burlington, ON L7N 3N4, Canada) was used for genomic DNA isolation. In brief, a single isolated colony was used to inoculate 10 ml of MRS broth and grown overnight at 37°C without shaking under anaerobic conditions. The following day, the cells were harvested and heat treated at 37°C in the presence of 20 μg of lysozyme in Tris- HCl and EDTA buffer (pH 8.0). After treatment with proteinase K, RNAse A was added and the cells were incubated for 10 min at room temperature. The resulting mix was centrifuged at 12,000 rpm for 20 min, and the supernatant was transferred to a GeneJET column. After two washes with wash buffer, the DNA was finally eluted with Tris buffer (pH 8.0).

### 16S rRNA Amplification, Sequence Analysis, and Alignment

The genomic DNA extracted as described above was used as the template for the PCR amplification of 16S rRNA using the bifido16S universal primers 357F/926R (357F 5′-CCTACGGGAGGCAGCAG-3′; 926R 5′-CCGTCAATTCMTTTRAGT-3′) and DyNAzyme II DNA polymerase (Thermo Scientific). The cycling parameters were: initial denaturation at 95°C for 5 min, followed by 35 cycles at 95°C for 30 s, 56°C for 45 s, and 72°C for 1 min, and a final extension at 72°C for 5 min. Each reaction was analyzed on 1% agarose gel. The resulting PCR products were subjected to DNA sequencing with an ABI 310 DNA sequencer (institutional facility). Sequence similarity was determined with the National Center for Biotechnology Information BLAST tool (NCBI).

## Results

**FIGURE 1:**
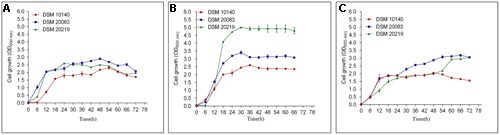
**Growth curves of DSM 10140 (*B. animalis* subspp. *lactis)*, DSM 20083 (*B. adolescentis*), DSM 20219 (*B. longum)* at different temperatures. (A)** 30°C, **(B)** 37°C, **(C)** 42°C.

The analysis of the 16S rRNA nucleotide sequences, Hsp60, and F6PPK showed distinct differences between the *Bifidobacterium* species. From the group three species were chosen for the study as they symbolize all the Bifidobacteria species. Certainly temperature does play a major part in the growth of Bifidobacteria. All together the three species [*B. animalis* subsp. *lactis* (DSM accession no. 10140), *B. adolescentis* (DSM accession no. 20083), and *B. longum* (DSM accession number: 20219)] grew well at 37°C reaching an OD_600_ of 3.0–5.0 after 66 h of culturing (**Figure 1B**). When the temperatures were reduced to 30°C the growth rates were drastically reduced (**Figure 1A**). The whole of the Bifidobacteria species were unable to come to an OD_600_ of 3.0 even after 72 h of growth. Nevertheless, considerable growth was observed for *B*. *adolescentis*. The remaining two Bifidobacteria grew very slowly, and an extended lag phase was discovered. When the growth curves were observed at an increased temperature of 42°C (**Figure 1C**) an outcome similar to that discovered at lower temperatures was observed (**Figure 1A**). Nevertheless, the cells did not obtain an OD_600_ above 3.0, even after 72 h of growth. These results indicate that 37°C may be an optimal temperature for the growth of these bacterial strains (**Figure 1B**). The growth curve studies further emphasize that *B. animalis* subsp. *lactis* is very slow growing (obtained an OD_600_ of 2.0–2.5), and it maintained this growth rate at different temperatures (30–42°C). *B. longum* showed the greatest growth rates at 37°C, and its growth rate was greater than either of the other strains. Strikingly, *B. adolescentis* grew faster at 37°C, reaching an OD_600_ of 3.0 in 30 h, a task which takes 48 h at 30°C and 72 h at 42°C.

Three different Bifidobacteria such as *Bifidobacterium adolescentis, B. animalis*, and *B. longum* was grown under different concentrations of oxygen and their growth curves analyzed. The results clearly demonstrated that as the concentration of oxygen in the system increased, the multiplication rate of the bacteria decreased and *vice versa* (data not shown). The different Bifidobacterial species displayed different resistance and susceptibility to oxygen (in the first generation). In the second generation, all the species grew aerobically, irrespective of the presence (of different concentrations) of oxygen. An oximetric study also showed that each *Bifidobacterium* species required different amounts of oxygen for growth, especially in the first generation. This further confirms that Bifidobacteria does show intolerance in the IInd generation, equally it is sensitive in the first generation (Beerens et al., 2000; Kawasaki et al., 2006; Ruiz et al., 2011).

**FIGURE 2:**
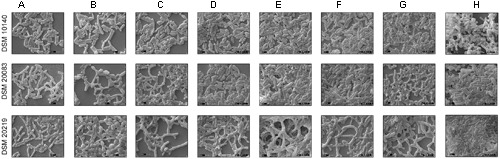
**Scanning electron microscopy (SEM) of strains DSM 10140 (*B. animalis* subspp. *lactis*), DSM 20083 (*B. adolescentis*), DSM 20219 (*B. longum)* with different conditions like (A)** 37°C, **(B)** 42°C, **(C)** media with alanine, aspartate, glutamate, serine, **(D)** media with alanine, **(E)** media with tryptophan, **(F)** media with asparagine, **(G)** media with 0.2 M sodium acetate, **(H)** media with 0.25 M sodium chloride.

To investigate the various morphologies of the Bifidobacterial species, they were subjected to various conditions and analyzed with SEM (**Figures 2A–H**). The experiments were performed at different temperatures (37, 42°C; **Figures 2A,B**), in different media (Supplementary Information), supplemented with an amino acid mixture containing alanine, aspartate, glutamate, and serine (**Figure 2C**), and also individually supplemented with alanine (**Figure 2D**), asparagine (**Figure 2F**), tryptophan (**Figure 2E**), or salts such as 0.2 M CH_3_COONa (**Figure 2G**) and 0.25 M NaCl (**Figure 2H**). The growth rate of the bacteria varied between species when the temperature was increased. All the species reached stationary phase after 36–48 h, at 37°C and after 42–60 h, at 42°C (**Figures 1B,C**). Rather than considering all 63 different species of *Bifidobacterium*, we selected species representative of the groups of Bottacini et al. (2014): *B. animalis* (DSM 10140), *B. adolescentis* (DSM 20083), and *B. longum* (DSM 20219) (Bottacini et al., 2014). We subjected them to different conditions, and observed any morphological changes with SEM. **Figures 2**, **10** clearly demonstrates the effects of each condition on the Bifidobacterial morphology. *Bifidobacterium adolescentis* (DSM 20083) metamorphosed from a bifid to a rod when grown on medium supplemented with alanine (A), asparagine (N), or 0.25 M NaCl. When *B. longum* (DSM 20219) was grown on medium supplemented with the four amino acids (Ala, Asp, Glu, and Ser), or separately with alanine (A) or asparagine (N), its morphology changed from elongated rods to bifids. However, *B. animalis.* subsp. *lactis* (DSM 10140) remained in the rod form, irrespective of its growth conditions.

### Supplementary Data to Understand the Other Bifidobacterial Species

**FIGURE 3:**
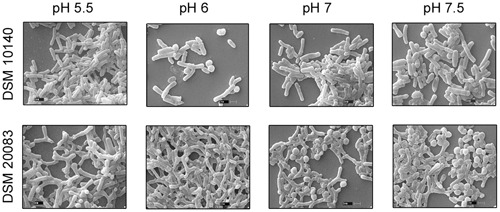
**Scanning electron microscopy of strains DSM 10140 (*B. animalis* subspp. *lactis*), DSM 20083 (*B. adolescentis*) in different pH condition**.

**FIGURE 4:**
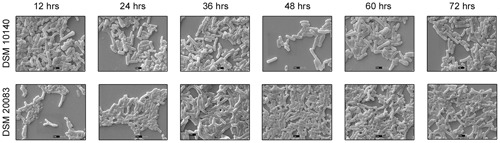
**Scanning electron microscopy of strains DSM 10140 (*B. animalis* subspp. *lactis*), DSM 20083 (*B. adolescentis*) in different time interval**.

**FIGURE 5:**
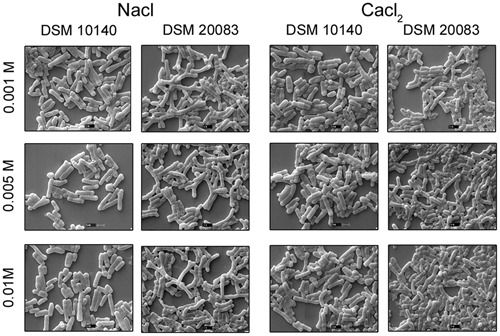
**Scanning electron microscopy of strain DSM 10140 (*B. animalis* subspp. *lactis*), and DSM 20083 (*B. adolescentis*) in different salts with varied concentration**.

**FIGURE 6:**
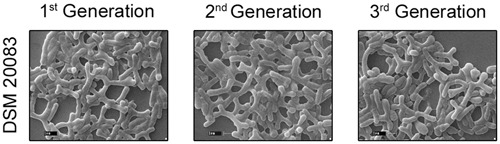
**Scanning electron microscopy of strain DSM 20083 (*B. adolescentis*) at three different generations**.

Another five species, *B. thermacidophilum* (DSM 15837), *B. infantis* (DSM 20088), *B. asteroides* (DSM 20089), *B. animalis* (DSM 20105), and *B. breve* (DSM 20213) was also subjected to the different conditions described above, and the results are shown in the **Supplementary Figure S1**. We noted a marked change in the morphology of *B. thermacidophilum* from rod- to bifid-shaped when grown on medium containing tryptophan. When *B. asteroides* was grown at 37°C, a mixed population of rod- and bifid-shaped bacteria were observed. Morphological changes were also observed when *B. breve* was grown with all four amino acids or with alanine alone, when it metamorphosed from rod- to bifid-shaped cells. *Bifidobacterium infantis* changed from rods to coccoids at 42°C. Almost all the other strains of *Bifidobacterium* maintained the rod shape under all the conditions tested, except as noted above. These findings confirm that *B. animalis* maintains its rod form irrespective of the conditions, implying that this morphology is an intrinsic characteristic. Therefore, *B. animalis* may be used as a reliable control when investigating the bifid nature of *B. adolescentis* (in MRS, at 37°C, and under anaerobic conditions). *B. adolescentis and B. animalis* (a control for rod shape) were exposed to other variable conditions, including various pH (**Figure 3**), for different growth periods (**Figure 4**), and in different concentrations of salt (**Figure 5**). The bifid shape was maintained by *B. adolescentis* under all the conditions tested, whereas the control, *B. animalis*, retained the rod form. The bifid shape of *B. adolescentis* were also retained when it was grown for three successive generations under normal conditions (**Figure 6**), in all of which the bifid shape was retained. This further affirms that the intrinsic bifid nature in the case of *B. adolescentis*, and rod shape in the case of *B. animalis*.

We also found that most *Bifidobacterium* species stably maintained their morphological (rod) shape, irrespective of the conditions. As shown in the **Figure 2**, all the *Bifidobacterium* species were intrinsically rod-shaped, except *B. adolescentis*. Few of them adopted the bifid structure, and only under stress conditions (**Figures 2C,D,F,G** *B. longum*; where C, D conditions corresponds to *B. breve* [shown in the Supplementary Information], E condition corresponds to *B. thermacidophilum* [shown in the Supplementary Information], and D, F, G conditions corresponds to *B. adolescentis*) [where C refers to the combination of four amino acids; D refer to alanine; E refers to tryptophan; F refers to asparagine; G refers to 0.2 M sodium acetate). However, *B. adolescentis* is intrinsically bifid-shaped and maintains its bifid morphology irrespective of the ambient conditions.

### *Caenorhabditis elegans* as a Model Organism for Pleomorphism Studies

**FIGURE 7:**
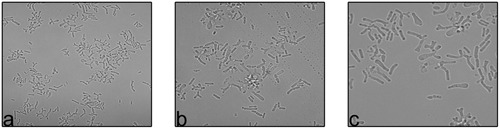
**Confocal images of *B. adolescentis* at different magnification (a)** Mag- 10x, **(b,c)** Mag- 60x.

To understand the bifid form of *B. adolescentis* and the forms of other *Bifidobacterium*, *C. elegans* was fed with different *Bifidobacterium* species and observed microscopically and by SEM. Based on chemotaxis study *B. adolescentis* and *B. longum* was considered for the study. Only *B. adolescentis* maintained the bifid shape in the gut of *C. elegans*. The *B. adolescentis*-fed *C. elegans* were dissected and centrifuged, and the supernatant was spread on an MRS agar plate. The numbers of bacteria were counted. The bacterial genomic DNA was isolated, and the 16S rRNA was amplified and analyzed, confirming that the isolated bacteria were *B*. *adolescentis*. The same culture was also subjected to an F6PPK assay, which confirmed the bacterial identity. The same bacteria were examined with confocal microscopy (**Figure 7**), which confirmed their bifid morphology. Bifidobacteria species other than *B. adolescentis* maintained their intrinsic rod shape in the digestive tracts of the worms, confirming that this is their intrinsic shape, and changes only under stress conditions.

### *Bifidobacterium* is the Preferred Food of *C. elegans*

**FIGURE 8:**
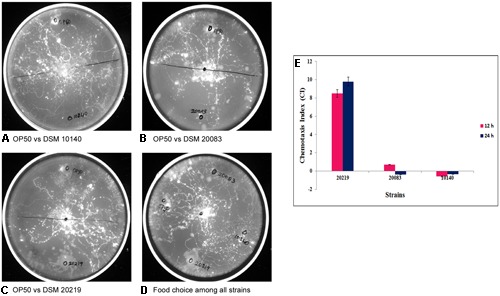
**Chemotaxis assay and chemotactic index (CI) for understanding the food of choice among the other strains by the *Caenorhabditis elegans* in comparison with control *Escherichia coli* OP50 vs. (A)** DSM 10140 **(B)** DSM 20083 **(C)** DSM 20219 and **(D)** in combination with all. **(E)** If CI = –1.0 represents complete preference for *E. coli* OP50. +1.0 represents complete preference for the test bacterium. 0.0 represents an equal distribution.

**FIGURE 9:**
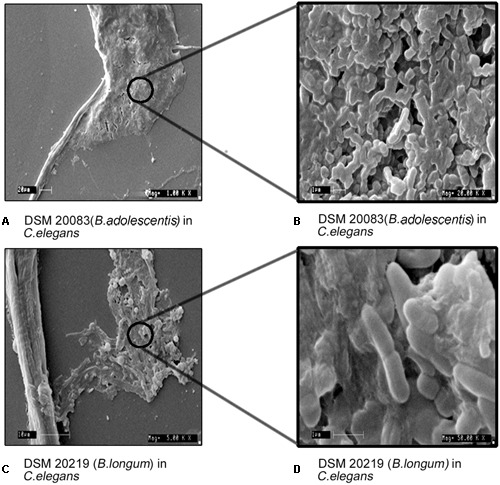
**Morphology of *B. adolescentis* (DSM 20083) and, *B. longum* (DSM 20219) in *C. elegans* with different magnification (Mag). (A)** DSM 20083 (*B. adolescentis*) in *C. elegans* Mag-1000x; **(B)** DSM 20083 (*B. adolescentis*) in *C. elegans* Mag-20,000x; **(C)** DSM 20219 (*B. longum*) in *C. elegans* Mag-5000x; **(D)** DSM 20219 (*B. longum*) in *C. elegans* Mag-50, 000x.

Experiments were performed to understand the ability of *C. elegans* to consume *Bifidobacterium*. The food chosen by *C. elegans* from among probiotic organisms and a control was estimated both qualitatively (**Figures 8A–D**) and quantitatively (**Figure 8E**), and represented as the CI. The CI for *B. longum* was 8.5 and 9.8 at 12 and 24 h, respectively, and was 0.7 for *B. adolescentis* at 12 h. *C. elegans* contained no *B. animalis* or *B. adolescentis* at 24 h, indicating that *C. elegans* had not selected them. To validate the bacterial morphology *in vivo*, nematodes that had consumed *B. longum* or *B. adolescentis* were fixed and examined with SEM to determine the shapes of the bacteria at different magnifications (**Figure 9**).

The results in the **Figure 8E** clearly show that the nematodes greatly preferred *Bifidobacterium* to *E. coli* OP50 as its staple food. *Caenorhabditi*s also expressed a preference among the three different *Bifidobacterium* species tested, so *C. elegans* may be the best model organism in which to investigate this polymorphism of *Bifidobacterium*. We assayed the preference of *C. elegans* for bacterial species *B. longum, B. adolescentis*, and *B. animalis*. The nematodes showed a preference for *B. longum* over *B. adolescentis* and *B. animalis* (**Figures 8A–D**). The histogram shows the CIs of wild-type *C. elegans* for bacterial strains *B. longum*, *B. adolescentis*, and *B. animalis* at 12 and 24 h (**Figure 8E**). The data are the means ± SD of three independent biological replicates.

The health benefits of probiotic microbes are only observed when they reach the colon, after passing through the small intestine (low pH), and large intestine (alkaline conditions). During their progress, they are exposed to very harsh conditions. To understand the effects on their morphology, these Bifidobacteria were subjected to different pH. When exposed to increasing pH (**Figure 3**), *B. adolescentis* slowly became coccoid, with a dramatic reduction in bifid bacteria. At pH 5.5, they maintained the bifid structure, but as the pH increased to 6, the bifid structure started to diminish, and at pH 7 and 7.5, the number of coccoid structures predominated, although no pointed elongated rods were observed (**Figure 3**). This phenomenon indicates that before it reaches its destination, the morphology of *B. adolescentis* changes frequently to accommodate the ambient conditions. Bacterial movement may be faster in the coccoid state, and this state may be favored when no adhesion is required. For adhesion, a larger and rougher bacterial surface area is required, and therefore a bifid shape is favored. Thus, *B. adolescentis* always maintained its bifid shape, unless it senses adverse conditions, when its morphology changes.

When Bifidobacterial species were grown in the presence of different salts (0.2 M sodium acetate or 0.25 M sodium chloride) (Kojima et al., 1968), SEM clearly showed that there were no dramatic changes in their morphologies (**Figures 2G,H**). A slight elongation of the rods was the only visible difference between salt-exposed *B. longum* and control *B. longum*. Importantly, the presence of lower concentration (0.001–0.01 M) NaCl did not affect the morphology of *B. adolescentis*, and its bifid structure did not change to the rod form. The bifid structure was observed at lower concentrations of salt, and as the concentration of salt increased, the bacteria assumed the rod shape. The rod structures started to diminish at a salt concentration of 0.005–0.01 M, whereas the bifid form started to shorten, until at 0.25 M salt, no bifid structures were seen (**Figure 2H**, condition of *B. adolescentis*).

Scanning electron microscopy also showed that *Bifidobacterium* other than *B. adolescentis* adopted the bifid form, but only very rarely, and only under stress conditions (**Figures 2B–H**). Therefore, it is clear that the bifid form is an intrinsic to *B. adolescentis*, but not to the other *Bifidobacterium* species, although a few Bifidobacterial species adopt the bifid form under stress conditions. Kojima et al. (1968) reported that changes to the bifid form occurred in different generations of *Bifidobacterium*, but our study clearly shows that the first, second and third generations of *B. adolescentis* maintained the bifid morphology (**Figure 6**). Dramatic changes in *B. adolescentis* were only observed when it was exposed to different pH (pH 5.5–7.5), when it adopted a coccoid shape (**Figure 3**). Therefore, the number of coccus-shaped bacteria was much higher depicted in the initiation of coccus formation, but reverted back to the bifid form. CaCl_2_ plays a significant role in the conversion of *Bifidobacterium* from the bifid shape to the rod form, suggesting the involvement of calmodulin or a similar molecule. This phenomenon was observed when *B. adolescentis* were subjected to different concentrations of CaCl_2_ (0.001–0.01 M). As the calcium concentration increased, the cells became rod-shaped, short, and bulging, with terminal buttons, and they also aggregated (**Figure 5**).

**FIGURE 10:**
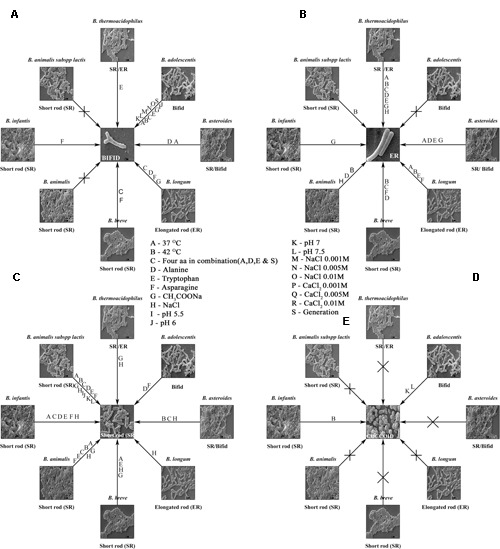
**Bifidobacterial species subjected to different conditions and their response are shown in the form of a wheel. (A–E)** Shows summary of the Bifidobacteria and their morphologies after exposure to different conditions. Different strains of Bifidobacteria and their inter changed morphologies after subjected to different conditions are depicted. **(A)** bifid shape, **(B)** elongated rod (ER), **(C)** short rod (SR), **(D)** cocci, **(E)** different conditions.

We examined *B. longum* in experiments similar to those described above because it bears an intrinsic elongated rod structure. As recorded in **Figure 2**, no dramatic changes were observed under different conditions, except when the cells were exposed to 42°C, their rod structures changed to extend elongated rods. At higher oxygen concentrations (10%), they aggregated and became elongated, with button/hyphal structures at their ends. When the concentration of oxygen was reduced, the hypha-like structures and cell aggregation, decreased (data not shown). A low concentration of calcium chloride (0.001 M) caused some defects, including cell shrinkage, the formation of wrinkles or holes, etc. As the levels increased from 0.005 to 0.01 M, the cells started out to change from rod-shaped to extensively elongated rods, as well as the bifid form. At all concentrations of CaCl_2_, the ratio of bifid-shaped *B. adolescentis* were drastically reduced in number (**Figure 5**, DSM 20083). Structural alterations of different Bifidobacterial species subjected to different conditions are summarized in **Figure 10**.

## Discussion

Many researchers have claimed that the *Bifidobacterium* species are strictly anaerobic, but no research has supported this theory. From our findings, we conclude that the *Bifidobacterium* species are microaerophilic because they can grow in the presence of a certain level of oxygen after the first generation. Weiss (1933) and Weiss and Rettger (1938) reported that *L. bifidus* and *L. acidophilus* is closely related, and are variants of the same species. This hypothesis was rejected for lack of evidence. Subsequently, Orla-Jensen (1943) reported that *B. bifidum* should be included in the order Actinomycetales. Weiss and Rettger later concluded that respect were two different kinds of *Bifidobacterium*: type 1 is aerobic and unbranched during primary isolation, and type 2 is anaerobic and branched. Weiss and Rettger (1938) proposed that all these organisms should be retained in *Lactobacillus*, but not in Actinomycetales. Established on the hypothesis of Weiss and Rettger, the Topley and Wilson’s Microbiology and Microbial Infection and Bergey’s Manual of Systematic Bacteriology adopted this classification. Orla-Jensen then proposed that the genus name should be converted to ‘*Corynebacterium.*’ It was reasoned that the use of different growth media for these bacteria may have caused the confusion concerning their nomenclature. These matters remain open today because most of these species are nevertheless brought up to as *Bifidobacterium*, although most are rod-formed. In 1967, using simple microscopy (not SEM), Masami Kojima found that cations play a significant role in the initiation of the bifid form. Nevertheless, we found that cations do not play a substantial role in bifid formation, although they induce the formation of elongated rods. To see the effects of NaCl on the bifidobacterial bifid and rod shapes, *B. animalis* subsp. *lactis* and *B. adolescentis* were grown in media containing 0.001–0.01 M salt (**Figure 5**) (Kojima et al., 1968). Both strains maintained their intrinsic morphologies, although at 0.25 M NaCl, *B. adolescentis* reverted from a bifid to a rod-shaped structure (**Figure 2H**). The *B. adolescentis* changes its intrinsic nature of bifid to rod when they are grown in bifid media (**Supplementary Figure S2**) and remains same in the presence of amino acid such as asparagine, tryptophan, alanine (individually) (**Supplementary Figure S3**) which is necessary for the branching of the bacteria (Husain et al., 1972).

These present studies showed that CaCl_2_ plays a major part in abolishing the bifid shape, which is the intrinsic morphology of *B. adolescentis*. Nevertheless, no significant conclusions were drawn from Kojima et al. (1970) research group involving the effects of cations on the bifid form of *Bifidobacterium*. Kojima et al. (1968) concluded that a calcium-free environment induces the bifid state, whereas the presence of calcium induces the bacilloid form. Yet, in the present study, CaCl_2_ induced the bacillus form of *B. adolsecentis*, despite its intrinsic bifid morphology. Therefore, although the bifid form is the intrinsic morphology of a few *Bifidobacterial* species, this is not absolute. CaCl_2_ may be considered a suppressor of the bifid state, supporting the proposition that there are calmodulins, calmodulin-like proteins in the *Bifidobacterium* species.

Our SEM analysis clearly shows that the growth medium (bifidobacteria broth or MRS) and different conditions (such as various temperatures, pH, and salts) do play a major part in the pleomorphic morphology of the *Bifidobacterium*. The presence and absence of amino acids did not (dramatically) affect their morphologies, converting the rod structure to the bifid form or to cocci, or the bifid form to the rod form. Not all *Bifidobacterium* have a bifid morphology, and only a very few are intrinsically bifid-shaped, such as *B. adolescentis.* It is well-demonstrated in this study that the intrinsic morphologies of these bacteria are rod-shaped, and all but a few do not adopt the bifid form, even after exposure to stress conditions. Thus, the rod structure is the intrinsic morphology of most bifidobacterial species. These data confirm the theory that the name ‘*Bifidobacterium*’ is a misnomer. Therefore, taxonomists must revisit this name by picking up more data in the near future.

As we expected, *Bifidobacterium* was the intellectual nourishment of choice for the eukaryotic model system, *C. elegans*, although the standard food offered *C. elegans* experimentally has been *E. coli* OP50. The nematodes tended to indicate a preference for *Bifidobacterium* over *E. coli* OP50. *C. elegans* also differentiates among the *Bifidobacterium*, as seen in **Figure 8**. Of the three *Bifidobacterium* species given as food, *C. elegans* preferred *B. longum* over *B. adolescentis* and *B. animalis*. The morphology of Bifidobacteria is intrinsically determined; the external factors don’t have anything to do with the same, example *B. adolescentis.* Equally it is the only strain out of eight tested in our study retained its bifid shape in the extremities.

## Author Contributions

D executed the idea; RK conceptualized, planned, designed, analyzed, Interpreted results and wrote the manuscript. SR and BK all the *C. elegans* work planning and execution.

## Conflict of Interest Statement

The authors declare that the research was conducted in the absence of any commercial or financial relationships that could be construed as a potential conflict of interest.

## Supplementary Material

FIGURE S1 **Scanning electron microscopy of strains DSM 15837 (*B. thermoacidophilus*), DSM 20088 (*B. infantis*), DSM 20089 (*B. asteroides*), DSM 20105 (*B. animalis*), DSM 20213 (*B. breve*) with different conditions like (A)** 37°C, **(B)** 42°C, **(C)** media with alanine, aspartate, glutamate, serine, **(D)** media with alanine, **(E)** media with tryptophan, **(F)** media with asparagine, media with 0.2 M sodium acetate, media with 0.25 M sodium chloride, **(G)** media with 0.2 M sodium acetate, **(H)** media with 0.25 M sodium chloride.

FIGURE S2 **Scanning electron microscopy of strains DSM 10140 (*B. animalis* subspp. *lactis*), DSM 20083 (*B. adolescentis*) in bifid media at time interval**.

FIGURE S3 **Scanning electron microscopy of strains DSM 10140 (*B. animalis* subspp. lactis), DSM 20083 (*B. adolescentis*) in bifid media with (A)** asparagine, **(B)** tryptophan, **(C)** alanine. Food choice assay: assaying the preference of *C. elegans* for bacterial species *B. longum, B. adolescentis*, and *B. animalis*. Nematodes showed a greater preference for *B. longum* than for *B. adolescentis*, or *B. animalis*. Chemotaxis assay: the histogram shows the chemotaxis index (CI) of wild-type *C. elegans* toward bacterial strains 20219, 20083, and 10140 after 12 and 24 h. Data are the means ± SD of three independent biological replicates.

## Abbreviations

bp: base pair
F6PPK: fructose-6-phosphate phosphoketolase
Ocins: antimicrobial peptides/proteins of *Lactobacillus, Bifidobacteria* and *Enterococcus* spp.
OD: optical density
*xfp*: xylulose-5-phosphate/fructose-6-phosphate phosphoketolase.
