# Supplementary figures and images for: Bifid Shape Is Intrinsic to Bifidobacterium adolescentis
Source: Front Microbiol. 2017 Mar 21;8:478. doi: 10.3389/fmicb.2017.00478 (PMC5359755; doi:10.3389/fmicb.2017.00478)

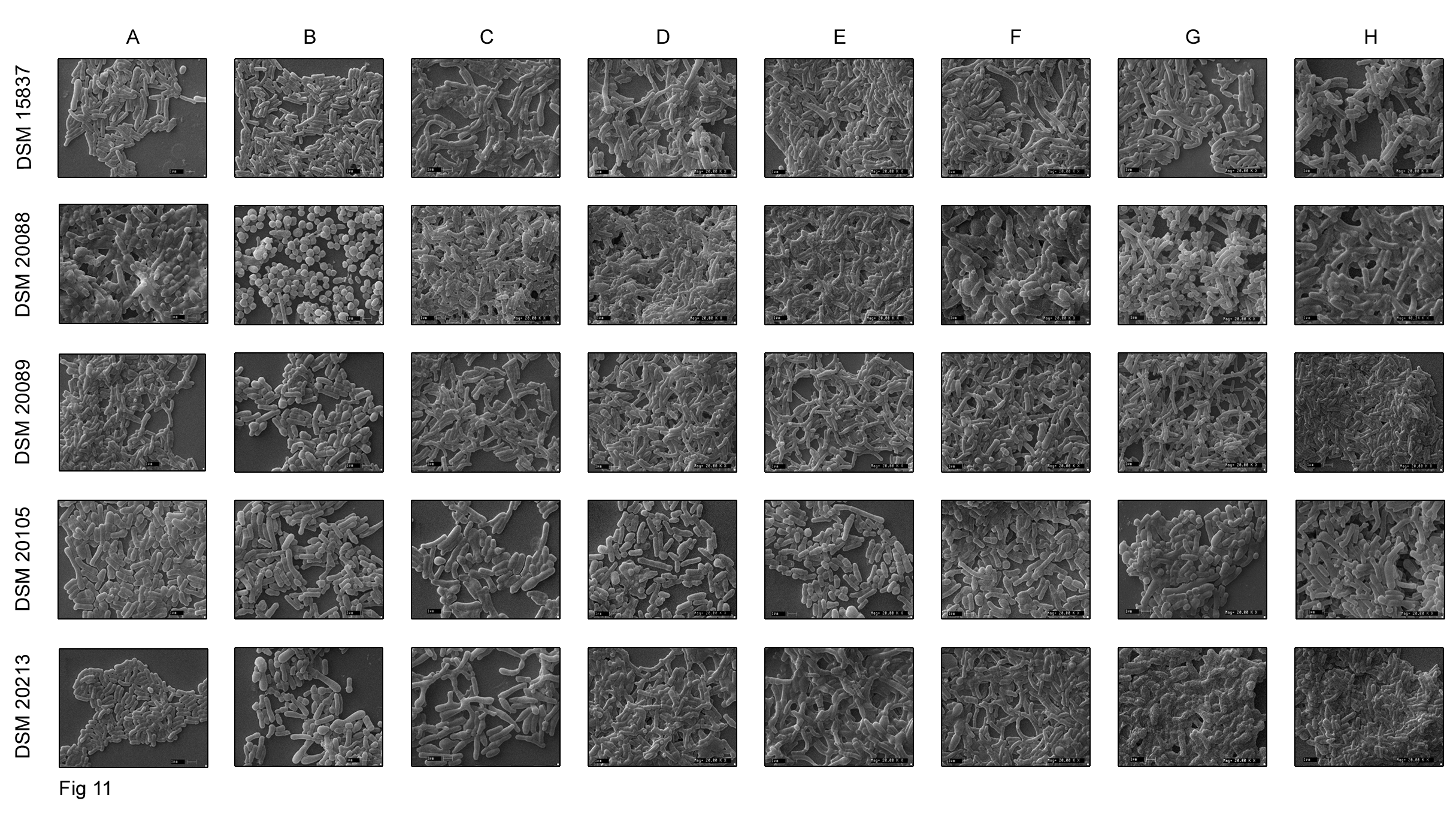

Supplement: FIGURE S1 — Scanning electron microscopy of strains DSM 15837 (B. thermoacidophilus), DSM 20088 (B. infantis), DSM 20089 (B. asteroides), DSM 20105 (B. animalis), DSM 20213 (B. breve) with different conditions like (A) 37°C, (B) 42°C, (C) media with alanine, aspartate, glutamate, serine, (D) media with alanine, (E) media with tryptophan, (F) media with asparagine, media with 0.2 M sodium acetate, media with 0.25 M sodium chloride, (G) media with 0.2 M sodium acetate, (H) media with 0.25 M sodium chloride. [file Image_1.tif]

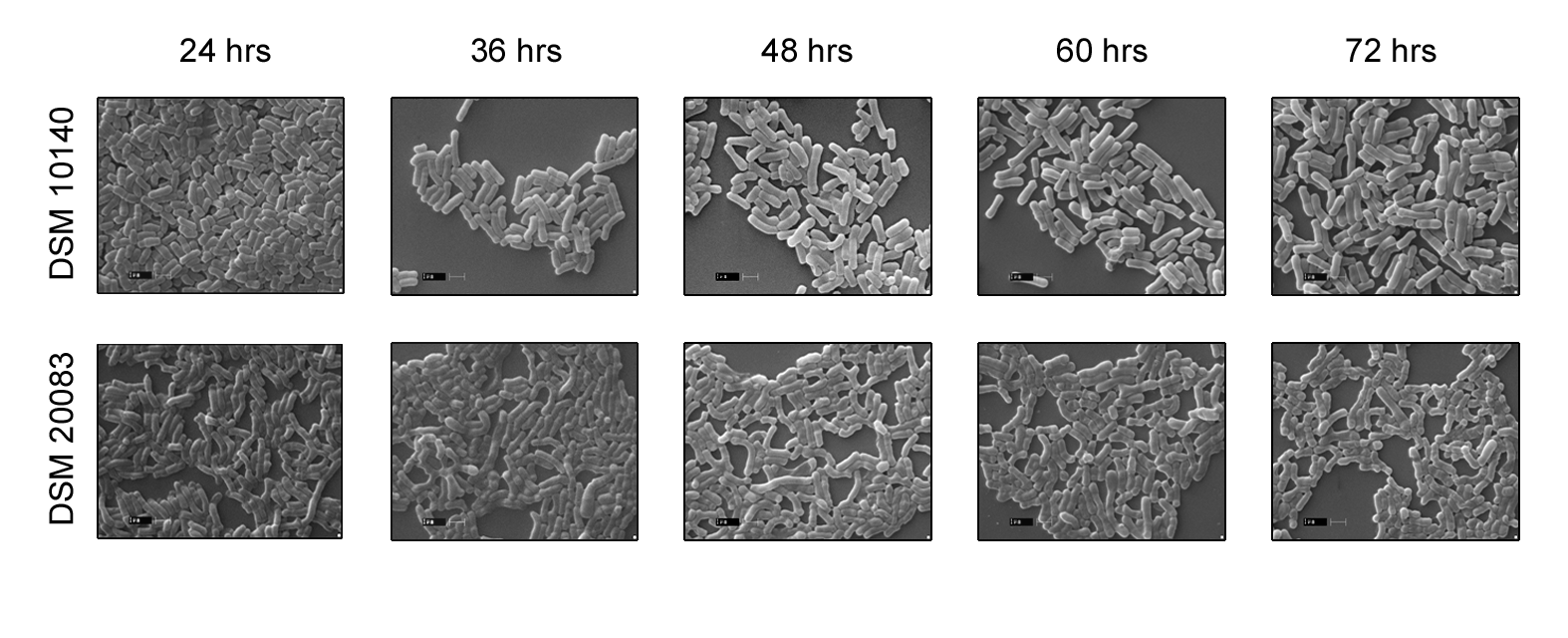

Supplement: FIGURE S2 — Scanning electron microscopy of strains DSM 10140 (B. animalis subspp. lactis), DSM 20083 (B. adolescentis) in bifid media at time interval. [file Image_2.tif]

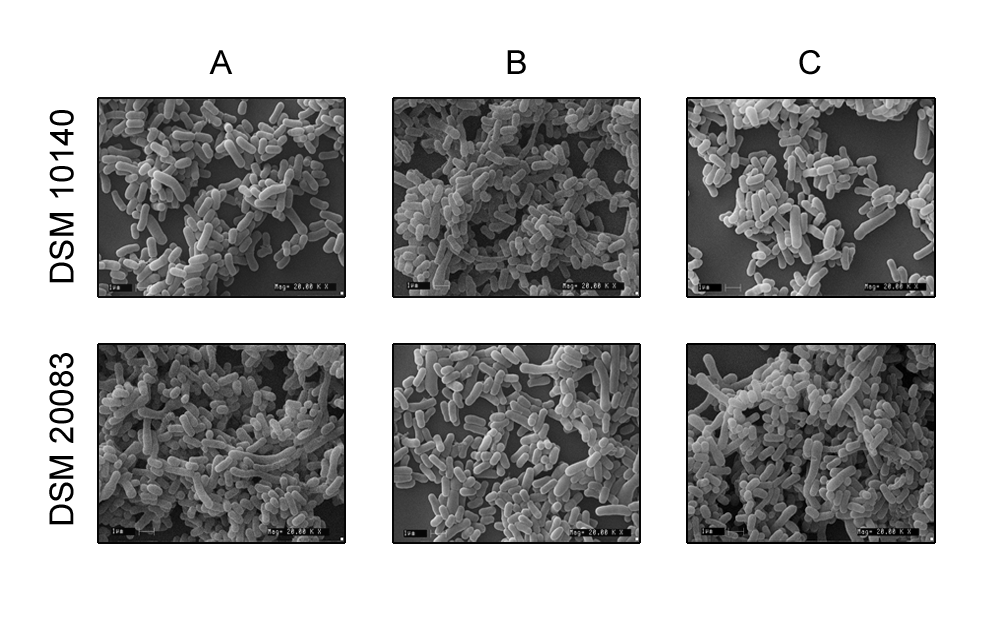

Supplement: FIGURE S3 — Scanning electron microscopy of strains DSM 10140 (B. animalis subspp. lactis), DSM 20083 (B. adolescentis) in bifid media with (A) asparagine, (B) tryptophan, (C) alanine. Food choice assay: assaying the preference of C. elegans for bacterial species B. longum, B. adolescentis, and B. animalis. Nematodes showed a greater preference for B. longum than for B. adolescentis, or B. animalis. Chemotaxis assay: the histogram shows the chemotaxis index (CI) of wild-type C. elegans toward bacterial strains 20219, 20083, and 10140 after 12 and 24 h. Data are the means ± SD of three independent biological replicates. [file Image_3.tif]
